# Grip strength and inflammatory biomarker profiles in very old adults

**DOI:** 10.1093/ageing/afx088

**Published:** 2017-05-25

**Authors:** Antoneta Granic, Karen Davies, Carmen Martin-Ruiz, Carol Jagger, Thomas B L Kirkwood, Thomas von Zglinicki, Avan Aihie Sayer

**Affiliations:** 1 Institute of Neuroscience, Newcastle University, Newcastle upon Tyne, UK; 2 NIHR Newcastle Biomedical Research Centre, Newcastle Universityand Newcastle upon Tyne Hospitals NHS Foundation Trust, Newcastle upon Tyne, UK; 3 Newcastle University Institute for Ageing, Newcastle upon Tyne, UK; 4 Newcastle University Ageing Biology Centre, Newcastle upon Tyne, UK; 5 Institute of Health & Society, Newcastle University, Newcastle upon Tyne, UK; 6 Institute for Cell and Molecular Biosciences, Newcastle University, Newcastle upon Tyne, UK; 7 MRC Lifecourse Epidemiology Unit, Faculty of Medicine, University of Southampton, Southampton, UK; 8 Academic Geriatric Medicine, University of Southampton, Southampton, UK

**Keywords:** older adults, cohort study, risk factor, grip strength, inflammatory biomarker, principal component analysis

## Abstract

**Background:**

weak grip strength (GS) and chronic inflammation have been implicated in the aetiology of sarcopenia in older adults. Given the interrelationships between inflammatory biomarkers, a summary variable may provide better insight into the relationship between inflammation and muscle strength. This approach has not been investigated in very old adults (aged ≥85) who are at highest risk of muscle weakness.

**Methods:**

we used mixed models to explore the prospective association between GS over 5 years in 845 participants in the Newcastle 85+ Study, and inflammatory components identified by principal component analysis (PCA). Cut-offs of ≤27 kg (men) and ≤16 (women) were used to define sub-cohorts with weak and normal GS at each assessment.

**Results:**

PCA identified three components, which explained 70% of the total variance in seven baseline biomarkers. Basal interleukin-6 (IL-6) and tumour necrosis factor (TNF-α) had the highest loadings on Component 1; stimulated IL-6 and TNF-α and homocysteine the highest on Component 2; high-sensitivity C-reactive protein (hsCRP) loaded positively and albumin negatively to Component 3. In adjusted mixed models, only Component 3 was associated with GS. One SD increase of Component 3 was associated with a 0.41 kg lower GS initially (*P* = 0.03) in all participants, but not with GS decline over time. Similar conclusions held for those in the weak and normal GS sub-cohorts.

**Conclusion:**

an inflammatory profile including hsCRP and albumin was independently associated with baseline GS. Future studies linking inflammatory profiles and muscle strength are needed to corroborate these findings in older adults.

## Introduction

Weak grip strength (GS)—an indicator of upper-body and overall muscle strength decline [1], has been recognised as a powerful predictor of multi-morbidity [2], disability [3] and mortality [4], and is a key component of frailty [5] and sarcopenia [1, 6] in young old (aged ≥65) and very old adults (aged ≥85). Because of the increasing public health burden associated with low muscle strength and poor physical functioning [7], it is important to identify risk factors and biological mechanisms that underlie the relationship between advancing age and decline in muscle strength.

A number of studies have demonstrated the role of chronic, low-grade inflammation in muscle strength decline [8]. An imbalance between pro- and anti-inflammatory biomarkers has been suggested to contribute to a detrimental catabolic effect of inflammation on aged myofibres, in the absence of an acute inflammatory response [9].

Both cross-sectional and longitudinal studies have shown the relationship between lower muscle strength and decline with higher plasma concentration of interleukin-6 (IL-6) and tumour necrosis factor alpha (TNF-α) and C-reactive protein (hsCRP) which were analysed either separately [10–15], or in combination [9, 16], or as a part of summary variable derived statistically to characterise inflammatory profile [17–19]. Assessing these biomarkers individually in relation to muscle strength and physical performance decline has been the commonest approach. For example, higher baseline concentrations of TNF-α were associated with a greater 5-year decline in GS and knee extensor strength in the Health, Aging and Body Composition (Health ABC) study of over 2000 participant aged 70–79 [10]. An increase in IL-6 concentration was associated with GS decline over 9 years in 901 participants in the Cardiovascular Health Study All Stars aged 85 at follow-up, irrespective of other relevant biological factors [16].

A few studies have gone further and taken into consideration the complexity of the inflammatory response and interdependence of individual inflammatory biomarkers. They have used multiple measures of inflammation to investigate the relationship between a summary variable [e.g. inflammatory index or components derived by principal component analysis (PCA)] and muscle strength. However, the results have been contradictory. In the Health ABC study, TNF-α and CRP-related components (derived by PCA from 8 inflammatory biomarkers) were inversely associated with GS [19], but higher inflammatory index (a combination of gender-adjusted tertiles of IL-6 and TNF-α) was not associated with worse GS and physical performance in older adults aged ≥80 living in Belgium [18]. Furthermore, little is known about the prospective inflammation–muscle strength relationship in the very old, who are at the highest risk of low muscle strength as well as subclinical inflammation [20].

Therefore, the aim of this study was to (i) identify inflammatory components from seven inflammatory biomarkers using PCA and (ii) investigate the association between identified components and change in GS over 5 years in the very old.

## Methods

### Study cohort

We included 845 participants from the Newcastle 85+ Study, a longitudinal study of individuals born in 1921 [mean age 85.49 (SD) = 0.44 years at baseline in 2006/07], and residing in Newcastle and North Tyneside region, United Kingdom, as described previously [21, 22]. Participants were followed up over 5 years from baseline [i.e. 1.5- (wave 2), 3- (wave 3) and 5-year follow-up (wave 4)]. At baseline, 813 (96.2%) participants had valid GS measurement, followed by 605 (71.6%) at wave 2, 452 (53.5%) at wave 3 and 294 (34.8%) at wave 4. Complete (baseline) data on 7 inflammatory biomarkers was available for 724 (85.7%) participants. The study was approved by the Newcastle and North Tyneside Local Research Ethics Committee One.

### Inflammatory biomarkers

Details about biomarker methodology, measurement and characteristics have been reported previously [20] (see Supplementary data, Appendix 1, available at *Age and Ageing* online). We included the following blood-based inflammatory biomarkers reported in the literature to be relevant for GS in later life: IL-6 (basal and stimulated; pg/mL), TNF-α (basal and stimulated; pg/mL), hsCRP (mg/L), homocysteine (HCY) (μmol/L) and albumin (g/L). All biomarkers except albumin were non-normally distributed, and were categorised in deciles to correct for marked positive skew. Albumin was divided in sextiles as the highest possible categorisation because of data granularity. Median and ranges of selected biomarkers are described in Supplementary data, Table S1, available at *Age and Ageing* online.

### GS measurement

GS was measured using a standardised protocol in standing position with a Takei hand dynamometer (Model A5401 digital 0–100 kg; Takei Scientific Instruments Co., Ltd., Niigata City, Japan) as described previously [23]. Two alternate measurements (in kg) for each hand were recorded, and the mean (*M*, SD) of four measurements for each participant for each wave was calculated [24]. GS data was normally distributed across the waves and was used as continuous variable in subsequent analyses.

### Other measures and potential confounders

Confounders for multivariable analysis included a set of sociodemographic, anthropometric, lifestyle and health-related factors previously determined to be associated with initial level and rate of change in GS in this [23] and other cohorts. All confounders and descriptive variables are defined in Supplementary data, Appendix 1, are available at *Age Ageing* online. The sociodemographic covariate was sex. Anthropometry included height, BMI and fat-free mass (FFM). Lifestyle included self-reported physical activity, and health-related variables comprised self-rated health, presence of depressive symptoms, multi-morbidity, arthritis in hands and intake of non-steroidal anti-inflammatory drugs [22]. To account for losses to follow-up (mostly death and withdrawal) over 5 years, we included an attrition variable (completed the study/dropped out) in the analyses of change [25]. All confounders were assessed at baseline, and height and FFM were centred to sex-specific mean.

We used cut-offs of ≤27 kg (men) and ≤16 kg (women), a strength of *T*-score ≤2.5 below sex-specific peak mean at age of 32 [26] to define a weak versus normal GS at baseline and follow-up, and to distinguish between the ‘weak’ and ‘normal’ GS sub-cohorts in longitudinal analysis of change in GS (for details see Supplementary data, Appendix 1, available at *Age and Ageing* online). We identified 1,607 weak and 557 normal GS observations over the study period.

For Statistics and sensitivity analysis, Supplementary data, Appendix 2, are available in *Age and Ageing* online.

## Results

### Inflammatory components

Biomarker descriptive statistics (median and range) and Spearman correlation coefficients (*r*) among pairs of seven inflammatory biomarkers are presented in Supplementary data, Table S1 and Table S2, are available in *Age and Ageing* online. Highest correlation (*r* ≥ 0.71) was observed between cytokines IL-6 and TNF-α (both basal and stimulated).

PCA with inflammatory biomarkers derived three components which explained 70% of the variance in the inflammatory variables: 34.9% (Component 1), 19.6% (Component 2) and 15.6% (Component 3) (Table 1). Communality coefficients were highest for basal IL-6 and basal TNF-α (0.86) which loaded the highest on Component 1 (‘Basal cytokines-related component’). Stimulated TNF-α, stimulated IL-6, and HCY loaded highest on Component 2 (‘Stimulated cytokines-related component’) and hsCRP and albumin loaded the highest onto Component 3 (‘hsCRP-related component’).

**Table 1.** Principal components statistics (eigenvalues, communalities and rotated component loadings)^a^ for three inflammatory components in the very old

Component	Eigenvalues	% Of variance		Biomarker	Communalities	Rotated component loadings		
			% Total variance			Component 1	Component 2	Component 3
Component 1	2.44	34.85		IL-6 stimulated	0.78		0.85	
Component 2	1.37	19.57		TNF-α stimulated	0.82		0.85	
Component 3	1.09	15.63		IL-6 basal	0.86	0.92		
			70.05	TNF-α basal	0.86	0.89		
				hsCRP	0.68			0.82
				HCY	0.25		0.44	
				Albumin	0.65			−0.80

IL-6, interleukin-6; TNF-α, tumour necrosis factor alpha; hsCRP, high-sensitivity C-reactive protein; HCY, homocysteine.

^a^Principal component analysis and orthogonal varimax rotation with Kaiser normalisation. A scree plot confirmed the presence of three independent inflammatory components. Loadings > 0.4 were used to identify biomarkers contributing to a component. Lower loadings were not reported.

### Characteristics of participants by inflammatory components

Raw GS data by tertiles of each inflammatory component across four waves, and baseline characteristics of participants by these tertiles are shown in Supplementary data, Table S3 and Table S4, are available in *Age and Ageing* online. Participants in Component 3 (‘hsCRP-related component’) differed significantly on key health and lifestyle characteristics across the tertiles. Specifically, participants in the highest tertile were the most likely to have fair/poor health (*P* < 0.001), to be obese (*P* = 0.001), the least physically active (*P* < 0.001), to have depressive symptoms (*P* = 0.001), dementia (*P* = 0.007), cardiovascular diseases (*P* = 0.006), metabolic syndrome (*P* = 0.02), the highest total cholesterol/HDL ratio (*P* < 0.001), and not to complete the study (*P* < 0.001) compared with participants in other tertiles.

### GS trajectories by inflammatory components

Significant associations with GS were found only for Component 3 (hsCRP-related component). Table 2 shows multivariable adjusted *β* coefficients (fixed effects) of Component 3 on GS initially, and the main effect of time on GS decline in the presence of important confounders and time × covariate interaction terms. In all models we observed a significant linear decline in GS of approximately −0.7 to 1 kg (entire cohort), −0.5 kg (normal GS sub-cohort) and −0.5 to −0.8 kg (weak GS sub-cohort) per year (all *P* ≤ 0.002). In Model 1, 1 SD increase in Component 3 score was associated with −1.05, −1.02 and −0.51 kg weaker GS (all *P* < 0.001) in the entire cohort, normal and weak GS sub-cohort, respectively. In Model 2, additional adjustment for important confounders and interaction terms attenuated the associations, but they remained significant in all groups. In Model 3, the association was sustained only in the normal GS sub-cohort after the inclusion of the attrition variable: 1 SD increase in Component 3 score was associated with −0.51 kg lower initial GS (*P* = 0.02). However, Component 3 was not associated with the rate of decline (slope) in GS over 5 years (data not shown).

**Table 2.** Multivariable adjusted β-coefficients of growth curve models for GS by Component 3 (hsCRP-related) over 5 years in the very old

Effects	Multivariable adjusted					
	Model 1		Model 2		Model 3	
	*β* (SE)^a^	*P*	*β* (SE)	*P*	*β* (SE)	*P*
*Entire cohort*						
GS initial status						
Intercept	17.76 (0.29)	<0.001	10.68 (0.70)	<0.001	10.49 (0.70)	<0.001
Component 3^b^	−1.05 (0.28)	<0.001	−0.41 (0.19)	0.03	−0.35 (0.19)	0.06
Decline^c^						
Time	−0.65 (0.14)	<0.001	−0.96 (0.20)	<0.001	−0.98 (0.20)	<0.001
Time^2^	−0.03 (0.03)	0.28	−0.30 (0.03)	0.26	−0.03 (0.03)	0.27
*Normal GS sub-cohort*^d^						
GS initial status						
Intercept	24.39 (0.46)	<0.001	16.39 (0.82)	<0.001	16.36 (0.83)	<0.001
Component 3^b^	−1.02 (0.47)	0.03	−0.51 (021)	0.02	−0.51 (0.21)	0.02
Decline^c^						
Time	−0.47 (0.08)	<0.001	−0.45 (0.071)	<0.001	−0.45 (0.07)	<0.001
*Weak GS sub-cohort*^d^						
GS initial status						
Intercept	15.41 (0.26)	< 0.001	10.70 (0.62)	<0.001	10.57 (0.62)	<0.001
Component 3^b^	−0.51 (0.24)	0.04	−0.31 (0.15)	0.03	−0.26 (0.15)	0.08
Decline^c^						
Time	−0.52 (0.17)	0.002	−0.76 (0.22)	0.001	−0.78 (0.22)	0.001
Time^2^	−0.02 (0.03)	0.14	−0.05 (0.03)	0.14	−0.05 (0.03)	0.14

FFM, fat-free mass; GS, grip strength; hsCRP, high-sensitivity C-reactive protein.

^a^*β*-coefficients (SE) are estimates of fixed effects using longitudinal grip strength data, and assess population averages in GS.

^b^Fixed effect of covariate estimated initial level and trajectory differences in GS as a function of the covariate.

^c^The main effect of time (i.e. Time and Time^2^) tested linear and non-linear change in GS across population over 5 years. Random effects included both intercept and slopes (linear change).

^d^Weak GS was defined as having a strength ≤27 kg in men, and ≤16 kg in women. This cut-off was used to identify participants with weak and normal GS across 4 measurements.

Model 1 includes a linear and quadratic trend of time (except in normal GS sub-cohort), and Component 3.

Model 2 is additionally adjusted for:

*Entire cohort*: sex, self-rated heath, anthropometry (height, FFM), physical activity, multi-morbidity and interaction terms (time × sex, time × physical activity).

*Normal grip strength sub-cohort*: sex, marital status, height, depressive symptoms and physical activity.

*Weak grip strength sub-cohort*: the same covariates and interaction terms as the entire cohort and for BMI (instead of FFM).

Model 3 is additionally adjusted for attrition variable.

The models include only significant predictors at first entry.

For the results for sensitivity analysis, Supplementary data, Appendix 3, are available at *Age and Ageing* online.

## Discussion

To the best of our knowledge, this is the first prospective evaluation of the relationship between summary variables of inflammation (produced by PCA) and GS decline in the very old (aged ≥85). Utilising data from the Newcastle 85+ Study, we found that three components could be used to summarise 7 inflammatory biomarkers (basal and stimulated IL-6 and TNF-α in PBMC, hsCRP, HCY and albumin) previously identified in the literature to be associated with low muscle strength (GS) in older adults. Only Component 3 (‘hsCRP-related component’) was inversely associated with GS initially but not with 5-year GS decline after adjustment for important confounders. The results suggest a role of the hsCRP-related but not the cytokine-related inflammatory component for muscle strength in very old adults.

Most studies to date have assessed the relationship between inflammation and physical performance in older adults using an individual biomarkers approach. They have reported an inverse association between baseline hsCRP, IL-6 and TNF-α (from serum or plasma) and muscle strength (GS) [11–15], and some evidence for their involvement in GS decline over time [9, 10, 12, 16]. A few considered alternative methods to create a latent variable or inflammatory index from multiple inflammatory biomarkers to account for their interrelationship [18, 19]. Only two included adults aged 80 and over, but reported conflicting results [15, 18]. Comparison between the studies employing an inflammatory profile approach in relation to muscle strength is difficult because of the differences in biomarker selection, methods used to derive summary measure of inflammation, physical performance tests used, and the population included. We are aware of only one study (Health ABC) that used PCA to create inflammatory components, and explored their cross-sectional relationship with muscle strength in older adults aged 70–79 [19]. A CRP-related component (characterised by the highest loading of serum CRP, IL-6 and plasminogen activator inhibitor-1) was associated with lower GS after adjustment for important confounders. Using a similar approach, we have also found the strongest association with a hsCRP-dominated component (Component 3) and worse initial GS (but not GS decline) in very old adults, particularly in those who maintained normal GS over 5 years [>27 kg (men), >16 kg (women)]. No association was found between cytokine-related components and GS, which could be attributed to the source of cytokines and the type of inflammation. Whilst other studies measured Il-6 and TNF-α in serum or plasma (systemic inflammation), we measured cytokines released from PBMC (either under no stimulation or following the cells activation). The negative results may suggest that in comparison with the more robust markers of chronic inflammation (Component 3), PBMC-released cytokines (Component 1) and the ability of PBMC to mount a response against infection (Component 2), play no or only a limited role in influencing muscle strength (GS) in very old adults.

Although data reduction analyses such as PCA makes no prior assumptions about the biological mechanisms underlying inflammation–muscle strength relationships, they may detect potentially unexplored interactions between biomarkers relevant for overall [27] and muscle health. Because we detected little or no association between individual biomarkers and GS prior to PCA (data not shown), the PCA approach may have strengthened the association between inflammation and muscle strength compared with an individual biomarker approach. However this needs to be confirmed in other studies involving the very old.

Component 3 was characterised by two dominant bipolar eigenvectors: (i) hsCRP, an acute phase protein produced by liver in response to elevated IL-6, has been the most consistently recognised predictor of cardiovascular events in several population-based studies [8] and (ii) (serum) albumin, a negative acute phase reactant, which decreases in older adults because of its increased catabolism and decreased synthesis during inflammation and malnutrition [28]. A synergistic and cumulative effect of these biomarkers may have exacerbated pathways contributing to greater muscle catabolism and reduced myogenesis [29], increased vascular pathology [8] and frailty [27]. Participants with higher levels of Component 3 had poorer health, and were more likely to drop out of the study, which may have underestimated the associations. Also, we did not detect any association between Component 3 and GS decline possibly because of loss of power in the data, and selection bias of very old ‘survivors’.

### Study limitations and strengths

This study has several limitations: (i) the results are observational and do not imply causality, and are affected by the choice of biomarkers used in PCA; (ii) the number of selected biomarkers was small, thus the results are not definitive and need to be repeated in other studies involving the very old; (iii) other biological mechanisms (e.g. oxidation) and biomarkers (e.g. cytokine receptors) may contribute to muscle strength decline, and uncontrolled factors may still explain the inflammation-GS relationship (e.g. malnutrition, medication duration and dosage); (iv) limited comparison with other studies, because we used PBMC-released cytokines; (v) inflammatory biomarkers at baseline may be poor predictors of change in GS; (vi) PCA is an exploratory technique, thus the utility of the individual biomarker approach should still be recognised; and (vii) limited generalisability of the results to the wider very old population. The strengths of the study include: (i) its prospective design, (ii) the novelty and robustness of the PCA approach in deriving inflammatory profiles, (iii) representativeness of the UK population and (iv) adjustment for several factors associated with muscle strength and inflammation.

### Future directions

We have provided preliminary evidence for the utility of the PCA approach in deriving inflammatory profiles from a set of inflammatory biomarkers to explore their relationship with muscle function in the very old. This approach and the results need to be corroborated in other prospective studies of older adults and should include a wider range of inflammatory biomarkers relevant for muscle function in later life [27].

In conclusion, we have identified three inflammatory biomarker profiles but only the one including hsCRP and albumin showed an association with baseline GS independent of key risk factors. These findings need to be corroborated in future studies involving the very old.

**Key points**

- We have investigated a summary measure of inflammation (produced by PCA) in relation to GS decline in very old adults.
- We identified three inflammatory biomarker profiles but only the one including hsCRP and albumin was related to baseline GS.
- Future studies linking inflammatory profiles and muscle strength are needed to corroborate these findings in older adults.

## Supplementary Material

Supplementary Data
