# Supplementary material for: Grip strength and inflammatory biomarker profiles in very old adults
Source: Age Ageing. 2017 May 25;46(6):976–82. doi: 10.1093/ageing/afx088 (PMC5860623; doi:10.1093/ageing/afx088)
Supplement: Supplementary Data [file afx088_aa-16-1042-file002.docx]

**Supplementary Data**

**Appendix 1**

**Methods**

*Inflammatory biomarkers*

Inflammatory biomarkers measured at baseline were: IL-6 (basal and stimulated; pg/mL), TNF-α (basal and stimulated; pg/mL), hsCRP (mg/L), homocysteine (HCY) (μmol/L), and albumin (g/L) (Table S1).The inflammatory response of the cytokines (IL-6 and TNF-α) was measured in the supernatant of peripheral blood mononuclear cells (PBMC) obtained from lithium heparin blood samples under basal conditions or following stimulation by lipopolysaccharide (Invitrogen Ultrapure LPS, Autogenbioclear, Nottingham, UK), and assessed by electrochemiluminescence on a 96-well Multi-SPOT Meso Scale Discovery assay with a SECTOR Imager 6000 (Meso Scale Diagnostics, LLC. Gaithersburg, MD, USA). Both basal and stimulated cytokines (IL-6 and TNF-α) released from PBMC under either no stimulation or upon cell activation were considered in the subsequent analysis—the latter measuring the ability of PBMC to respond to infection and increase the inflammatory load upon aged muscle.

hsCRP (Dade Behring CardioPhase hsCRP immunoassay), HCY, and albumin were assessed at the Newcastle Royal Victoria Infirmary (RVI) biochemical laboratory, and biomarkers affected by fasting status and time of the day of collection were coded as missing (<5% of the sample). The assay coefficient of variation (intra-assay/inter-assay) were reported by manufacturer as 8.11%/14.2% for basal IL-6, 8.47%/15.4% for basal TNF-α, 6.26%/11.8% for stimulated IL-6, and 8.21%/13.8% for stimulated TNF-α, and as 3.50%/7.83% for hsCRP, 4.53%/6.53% for HCY, and 1.4%/<3% for albumin by RVI laboratory, respectively.

*Description of confounders and variables used in descriptive analysis*

The following sociodemographic, anthropometric, lifestyle and health-related confounders were included in the linear mixed models: (i) sociodemographic were sex, marital status (single / widowed, separated, divorced / married); (ii) anthropometric were height (calculated from demi-span formula in cm), BMI calculated as kg weight/m^2^ height and categorised as <18.5 (underweight) / >18.5<25 (normal) / >25<30 (overweight) / 30 (obese), and fat-free mass (FFM) estimated by bioimpedance using a Tanita-305 body-fat bioimpedance instrument, (Tanita Corp., Tokyo, Japan); (iii) lifestyle included self-reported physical activity [low (score 0-1) / moderate (2-6) / high (7-18)], which was derived from a physical activity questionnaire and based on frequency and intensity of physical activity performed per week [26], and (iv) health-related were self-rated health (excellent or very good / good / fair or poor), presence of depressive symptoms [none (score 0-5) / mild or moderate (6-7) / severe (8-15)] assessed by the Geriatric Depression Scale (GD-15) [22], and multimorbidity (continuous) evaluated from the general practice records review (GPrr) (chronic diseases included cardiovascular diseases [hypertension, cardiac disease], respiratory disease, cerebrovascular disease, diabetes, arthritis and cancer) [22]. Additional health-related confounders for sensitivity analyses were: the presence arthritis in hands (in any or both hands, yes / no), and intake of non-steroidal anti-inflammatory drugs (yes /no).

Descriptive characteristics of the cohort and analytic sample also included : (i) total number of medication (continuous); (ii) number of cardiovascular diseases (none / 1-2 / 3-4) (from the GPrr: hypertension, ischaemic heart disease, heart failure, atrial fibrillation); (iii) peripheral vascular disease (yes / no); (iv) metabolic syndrome (yes / no) (defined according to the National Cholesterol Education Program Adult Treatment Panel III criteria); (v) lipid profile including total cholesterol (mmol/L; continuous), low density lipoprotein (LDL) (mmol/L; continuous), high density lipoprotein (HDL) (mmol/L; continuous), total cholesterol/HDL ratio (continuous), and (vi) waist circumference (cm; continuous).

*Defining weak versus normal grip strength*

We defined weak GS as a strength of T-score equal or less than 2.5 below sex-specific peak mean at age of 32 (≤27 kg in men, and ≤16 kg in women) as described previously [26]. Briefly, a T-score for GS was calculated for each participant at baseline and follow-up and expressed as a multiple of the number of standard deviations (SD) below the peak (sex-specific) mean value encountered at young age or 51.9 (9.9) kg in men and 31.4 (6.1) kg in women at age of 32. A T-score of equal or less than -2.5 is used widely in the diagnosis of sarcopenia and osteoporosis.

**Appendix 2**

**Statistics**

*Principal Component Analysis*

We used PCA with a Varimax rotation to reduce 7 inflammatory biomarkers into a smaller set of inflammatory components. A three-component solution was determined based on eigenvalues >1, a scree plot, and the interpretability of the components. Variable loadings ≥0.4 were determined as significantly contributing to the component structure and lower loadings were omitted (Table 1). To determine the robustness of the components solution we inspected the correlation matrix (Supplementary data, Table S2) and diagonal elements in the anti-image correlation matrix (for all inflammatory biomarkers r ≥0.5, details not shown), Bartlett’s test of sphericity, and Kaiser-Meyer-Olkin measure of sample adequacy. A Varimax rotation with Kaiser Normalization (orthogonal transformation) was used to improve the interpretability of inflammatory components. The nomenclature for the components was given based on biomarkers with the highest loading in each component. We calculated principal components scores (continuous) using the Anderson-Rubin method, which were then standardised to yield a sample mean of 0 and SD of 1, and also categorised in tertiles.

*Descriptive statistics*

We calculated unadjusted GS measurements (M, SD) at baseline and follow-up by tertiles of each inflammatory component. Participants were compared on key sociodemographic, lifestyle, and health-related measures by inflammatory component tertiles using ANOVA for normally distributed, Kruskal-Wallis test for non-normally distributed variables, and Chi-square test for categorical variables.

*Linear mixed models*

We employed linear mixed models to examine the association between inflammatory components and initial level and rate of change in GS over 5 years in all participants (the ‘entire cohort’ thereafter), and in the weak and normal GS sub-cohorts selected as described earlier. We used time as a continuous variable to test for both linear and non-linear (i.e. quadratic) change in GS, and fitted a series of growth curve models with predictors determined to be associated with GS decline [23] and significant inflammatory components (continuous).

Linear mixed models allow simultaneous examination of individual trajectory of change (within-person variability or random effects at Level 1), and population averages (between-person variability or fixed effects at Level 2) by using all available measurement and including participants with incomplete data. At Level 1, the models evaluate how GS changes over 5 years for each participant, and at Level 2 the models determine the effect of covariates on GS (initially and over time).

Model 1 included a linear and quadratic trend of time (in the entire cohort and weak GS sub-cohort) and inflammatory component. In the entire cohort, Model 2 was additionally adjusted for sex, anthropometry (height and FFM), physical activity, self-rated health, multi-morbidity, and interaction terms (time*sex, time*physical activity). In the normal GS sub-cohort, Model 2 included sex, marital status, height, depressive symptoms, and physical activity. In the weak GS sub-cohort, Model 2 was additionally adjusted for the same covariates and interaction terms as in the entire cohort, except for BMI instead of FFM. Model 3 further included attrition variable in all groups.

All covariates were time-invariant (baseline). For all mixed models, we employed the SPSS MIXED procedure (SPSS, 2002) with the ‘REPEATED’ command, random intercepts and slopes, and scaled identity and unstructured covariance matrix at Level 1 and level 2, respectively.

*Interpretation of β coefficients*

Negative *β* coefficients (estimates) indicated weaker GS or decline. *β* coefficients for time represented annual linear change, and Time^2^ tested additional non-linear change (acceleration or deceleration) in GS. *β* coefficients for predictors captured their effect on GS at baseline (intercept). *β* coefficients for interaction terms (time*covariate) tested whether GS slopes varied by the covariate over time.

*Sensitivity analysis*

We used student t-test, Mann-Whitney, and Chi-square tests to compare 121 participants without inflammatory profile to those considered in the study (*n* = 724) across key variables.

To confirm the PCA component solution, we categorised all biomarkers in quartiles. Additionally, we selected 70% of random cases (*n* = 512) from 724 participants with complete biomarker data categorised in deciles (except albumin in sextiles), and repeated the PCA. Mixed models were repeated in men and women. Model 3 was additionally adjusted for the presence arthritis in hands, and intake of non-steroidal anti-inflammatory drugs.

All statistics were conducted using IBM SPSS (V.21; IBM Corporation, Armonk, NY, USA).

**Appendix 3**

**Results for sensitivity analysis**

Participants without an inflammatory profile at baseline were more likely to be women (*P* = 0.001), not to drink alcohol (*P* = 0.001), and not to complete the study (*P* = 0.003) compared to those with the inflammatory profile. Those in the analytic sample were less likely to fall (*P* = 0.001) and were more physically active (*P* = 0.001), but no other differences were observed (details not shown).

The PCA three-component solution was confirmed with all biomarkers categorised in quartiles, and in a random sample of 70% participants (details not shown). For example, we derived three components with comparable eigenvalues and the same factor loadings with inflammatory biomarkers categorised in quartiles (Component 1: 2.35, Component 2: 1.35, and Component 3: 1.03 explaining 68.7% variance in data).

The findings of mixed models were not changed after additional adjustment for the intake of anti-inflammatory drugs, and arthritis in hands in all groups. In sex-stratified analysis, initially significant associations between Component 3 and GS at baseline in both men and women were explained by other predictors (e.g. physical activity, FFM, self-rated health and height) (details not shown).

**Supplementary Tables**

**Table S1.** Descriptive statistics for selected baseline inflammatory biomarkers in the Newcastle 85+ Study

___________________________________________________________________________

Biomarker^a^ (unit) Median (range)

___________________________________________________________________________

IL-6, basal (pg/mL) 16.0 (0-40178.2)

IL-6, post-stimulation (pg/mL) 2159.5 (134.8-41899.7)

TNF-α, basal (pg/mL) 3.4 (0-2369.1)

TNF-α, post-stimulation (pg/ml) 491.2 (3.6-37217.1)

hsCRP (mg/mL) 2.6 (0.2-166.0)

Albumin (g/L) 40.0 (24.0-47.0)

HCY (μmol/L) 16.7 (7.3-119.4)

___________________________________________________________________________

hsCRP, high sensitivity C-reactive protein, HCY, homocysteine, IL-6, interleukine-6, TNF-α, tumor necrosis factor alpha.

^a^All biomarkers except albumin were categorized in deciles, and used in principal component analysis. Albumin was categorized in sextiles because of data granularity (assessed by SPSS visual binning).

**Table S2.** Correlation coefficients (*r*) between seven baseline inflammatory biomarkers^a^ (*n* = 724) in the Newcastle 85+ Study

____________________________________________________________________________________________________________________

Correlation coefficient IL-6 stimulated TNF-α stimulated IL6 basal TNF-α basal hsCRP HCY Albumin
____________________________________________________________________________________________________________________

IL-6, stimulated (pg/mL) **1.0** 0.71 0.27 0.34 0.11 0.13 -0.06

*P*-value <0.001 <0.001 <0.001 0.003 <0.001 0.14

TNF-α, stimulated (pg/mL) **1.0** 0.2 0.48 0.02 0.11 0.02

*P*-value <0.001 <0.001 0.63 0.002 0.64

IL-6, basal (pg/mL) **1.00** 0.73 0.15 0.06 -0.08

*P*-value <0.001 <0.001 0.11 0.04

TNF-α, basal (pg/mL) **1.0** 0.14 0.1 -0.06

*P*-value <0.001 0.006 0.09

hsCRP (mg/L) **1.0** 0.11 -0.37

*P*-value 0.004 <0.001

HCY (μmol/L) **1.0** -0.005

*P*-value 0.89

Albumin (g/L) **1.0**

*P*-value

____________________________________________________________________________________________________________________

hsCRP, high sensitivity C-reactive protein, HCY, homocysteine, IL-6, interleukine-6, TNF-α, tumor necrosis factor alpha.

^a^All inflammatory biomarkers except albumin were categorised in deciles. Albumin was categorised in sextiles. Spearman correlation coefficients (*r*).

**Table S3.** Unadjusted grip strength measurement (mean, SD) across four waves by tertiles of inflammatory components scores

Grip strength measurement (kg) Component Tertile 1 *n* Tertile 2 *n* Tertile 3 *n*

____________________________________________________________________________________________________________________

Component 1: Basal cytokines-related component

Baseline 18.67 (7.68) 237 16.99 (7.73) 233 17.97 (7.84) 235

1.5-year follow-up (wave 2) 17.24 (7.52) 174 16.58 (7.47) 183 17.36 (8.64) 188

3-year follow-up (wave 3) 16.59 (6.54) 138 15.89 (6.51) 128 17.13 (8.11) 139

5-year follow-up (wave 4) 15.15 (6.54) 85 14.11 (6.41) 93 15.77 (8.19) 89

Component 2: Stimulated cytokines-related component

Baseline 17.11 (7.27) 234 17.60 (7.81) 234 18.91 (8.12) 237

1.5-year follow-up 16.84 (7.24) 182 16.44 (8.25) 180 17.90 (8.14) 183

3-year follow-up 16.34 (6.72) 140 16.50 (7.75) 130 16.83 (7.59) 135

5-year follow-up 14.44 (6.59) 99 15.29 (7.65) 80 15.35 (7.79) 88

Component 3: hsCRP-related component

Baseline 18.25 (7.84) 241 18.54 (7.66) 236 16.81 (7.72) 288

1.5-year follow-up 17.61 (7.98) 204 17.24 (7.46) 182 16.16 (8.26) 159

3-year follow-up 16.38 (7.88) 159 16.72 (6.92) 140 16.59 (7.08) 106

5-year follow-up 14.72 (7.42) 106 15.13 (7.13) 102 15.26 (6.55) 59

____________________________________________________________________________________________________________________

hsCRP, high sensitivity C-reactive protein

**Table S4.** Characteristics of participants by inflammatory components tertiles in the Newcastle 85+ Study

___________________________________________________________________________________________________________________

Characteristics All participants Tertile 1 Tertile 2 Tertile 3 *P*

______________________________________________________________________________________________________________________________

*Component 1: Basal cytokines-related component*

*n*  845 242 241 241

Sex, men % (n) 37.8 (319) 42.1 (102) 35.8 (86) 42.1 (102) 0.26

Occupational class 0.42

routine/manual 52.0 (415) 53.5 (122) 50.7 (116) 47.6 (111)

intermediate 14.2 (113) 16.2 (37) 13.5 (31) 13.7 (32)

higher managerial/administrative 33.8 (270) 30.3 (69) 35.8 (82) 38.6 (90)

Marital status % (n) 0.16

single 8.2 (69) 6.7 (16) 10.4 (25) 9.1 (22)

widowed/separated/divorced 61.7 (519) 60.0 (144) 64.2 (154) 57.0 (138)

married 30.1 (253) 33.3 (80) 25.4 (61) 33.9 (82)

Self-rated health % (n) 0.84

excellent/very good 40.0 (330) 41.8 (100) 39.6 (93) 40.6 (97)

good 37.6 (310) 37.7 (90) 38.3 (90) 36.8 (88)

fair/poor 22.3 (184) 20.5 (49) 22.1 (52) 22.6 (54)

BMI % (n) 0.11

<18.5 6.3 (47) 6.2 (14) 7.2 (16) 7.1 (16)

>18.5<25 51.3 (382) 58.6 (133) 50.7 (112) 46.5 (105)

>25<30 32.6 (243) 27.3 (62) 32.6 (72) 35.0 (79)

>30 9.8 (73) 7.9 (18) 9.5 (21) 11.5 (26)

Fat mass, kg mean (SD) 18.87 (7.74) 18.11 (7.30) 18.87 (7.69) 19.28 (8.24) 0.36

Fat-free mass, kg mean (SD) 45.17 (8.88) 44.98 (8.82) 44.96 (9.19) 45.96 (9.15) 0.4

Height, cm 161.10 (7.86) 161.92 (7.90) 160.84 (7.29) 161.76 (7.69) 0.3

Waist circumference, cm mean (SD) 90.99 (12.11) 89.74 (11.45) 90.45 (12.53) 92.61 (12.23) 0.03

GDS % (n) 0.29

no depression 78.9 (607) 81.7 (188) 76.2 (173) 77.2 (176)

mild depression 12.7 (98) 11.3 (26) 13.2 (30) 14.0 (32)

severe depression 8.3 (64) 7.0 (16) 10.6 (24) 8.8 (20)

Dementia, yes % (n) 8.8 (74) 5.8 (14) 9.2 (22) 7.9 (19) 0.37

Arthritis in hands, yes % (n) 7.3 (60) 33.3 (16) 41.7 (20) 25.0 (12) 0.35

Cardiovascular diseases % (n) 0.58

none (0) 25.4 (215) 28.9 (70) 22.5 (54) 24.4 (59)

1-2 65.4 (553) 62.4 (151) 67.5 (162) 66.5 (161)

3-4 9.1 (77) 8.7 (21) 10.0 (24) 9.1 (22)

Peripheral vascular diseases, yes % (n) 7.0 (59) 7.9 (19) 6.7 (16) 7.4 (18) 0.88

Metabolic syndrome, yes % (n) 27.4 (214) 26.4 (64) 25.8 (62) 29.8 (72) 0.58

Multi-morbidity, mean (SD) 2.26 (1.22) 2.13 (1.20) 2.39 (1.31) 2.29 (1.20) 0.06

Lipid profile

Total cholesterol, mmol/L mean (SD) 4.85 (1.23) 4.99 (1.19) 4.93 (1.36) 4.71 (1.14) 0.04

LDL cholesterol, mmol/L mean (SD) 2.71 (1.04) 2.83 (1.03) 2.76 (1.13) 2.59 (0.97) 0.03

HDL cholesterol, mmol/L mean (SD) 1.50 (0.41) 1.50 (0.39) 1.53 (0.43) 1.49 (0.41) 0.4

Total cholesterol/HDL ratio 3.37 (0.96) 3.46 (1.01) 3.33 (0.90) 3.34 (0.99) 0.25

Intake of NSAIDs, yes % (n) 4.7 (40) 25.8 (8) 41.9 (13) 32.3 (10) 0.51

Total number of medication, mean (SD) 6.37 (3.83) 6.01 (3.60) 6.65 (4.17) 6.40 (3.78) 0.19

Physical activity % (n) 0.006

low 23.3 (187) 19.6 (47) 23.1 (55) 23.3 (56)

medium 43.0 (349) 35.8 (86) 48.3 (115) 42.9 (103)

high 33.7 (274) 41.8 (107) 26.6 (68) 31.6 (81)

Smoking status % (n) 0.3

never smoker 35.8 (301) 33.3 (80) 32.1 (77) 38.0 (92)

current smoker 5.7 (480) 7.1 (17) 5.8 (14) 3.3 (8)

former smoker 58.5 (491) 59.6 (143) 62.1 (149) 58.7 (142)

Current alcohol intake, yes % (n) 59.5 (488) 59.2 (142) 60.8 (146) 65.0 (156) 0.4

Attrition over 5 years % (n) 0.54

completed the study 40.6 (343) 40.1 (97) 42.9 (103) 45.0 (109)

dropped out 59.4 (502) 59.9 (145) 57.1 (137) 55.0 (133)

_____________________________________________________________________________________________________________________________ *Component 2: Stimulated cytokines-related component*

*n* 242 241 241

Sex, men % (n) 34.0 (82) 39.7 (96) 46.5 (112) 0.02

Occupational class 0.08

routine/manual 44.3 (101) 49.8 (114) 57.5 (134)

intermediate 17.1 (39) 14.4 (33) 12.0 (28)

higher managerial/administrative 36.5 (88) 34.0 (82) 29.5 (71)

Marital status % (n) 0.27

single 10.4 (25) 10.0 (24) 5.8 (14)

widowed/separated/divorced 57.9 (139) 64.2 (150) 57.0 (147)

married 31.7 (76) 27.8 (67) 33.2 (80)

Self-rated health % (n) 0.22

excellent/very good 46.6 (110) 38.5 (92) 37.0 (88)

good 31.8 (75) 36.4 (87) 44.5 (106)

fair/poor 21.6 (51) 25.1 (60) 18.5 (44)

BMI % (n) 0.02

<18.5 7.7 (17) 6.6 (15) 6.2 (14)

>18.5<25 57.5 (127) 52.9 (120) 45.6 (103)

>25<30 27.6 (61) 30.0 (68) 37.2 (84)

>30 7.2 (16) 10.6 (24) 11.1 (25)

Fat mass, kg mean (SD) 17.90 (7.52) 18.47 (7.97) 19.88 (7.65) 0.02

Fat-free mass, kg mean (SD) 43.65 (8.24) 45.30 (9.13) 46.94 (9.48) 0.001

Height, cm 160.79 (7.61) 161.14 (7.42) 162.61 (7.79) 0.02

Waist circumference, cm mean (SD) 88.02 (11.7) 91.32 (11.72) 93.41 (12.35) <0.001

GDS % (n) 0.37

no depression 79.9 (183) 75.8 (172) 79.5 (182)

mild depression 14.0 (32) 11.9 (27) 12.7 (29)

severe depression 6.1 (14) 12.3 (28) 7.9 (18)

Dementia, yes % (n) 7.9 (19) 7.4 (18) 7.5 (18) 0.98

Arthritis in hands, yes % (n) 35.4 (17) 37.5 (18) 27.1 (13) 0.62

Cardiovascular diseases % (n) 0.05

none (0) 29.5 (71) 27.7 (67) 18.7 (45)

1-2 63.1 (152) 63.6 (154) 69.7 (168)

3-4 7.5 (18) 8.7 (21) 11.6 (28)

Peripheral vascular diseases, yes % (n) 5.4 (13) 7.0 (17) 9.5 (23) 0.21

Metabolic syndrome, yes % (n) 22.0 (53) 26.4 (64) 33.6 (81) 0.02

Multi-morbidity, mean (SD) 2.12 (1.20) 2.25 (1.17) 2.44 (1.33) 0.02

Lipid profile

Total cholesterol, mmol/L mean (SD) 5.07 (1.23) 4.87 (1.26) 4.68 (1.19) 0.003

LDL cholesterol, mmol/L mean (SD) 2.88 (1.08) 2.71 (1.06) 2.58 (0.99) 0.007

HDL cholesterol, mmol/L mean (SD) 1.56 (0.41) 1.53 (0.43) 1.43 (0.37) 0.001

Total cholesterol/HDL ratio 3.40 (0.99) 3.31 (0.92) 3.42 (0.99) 0.38

Intake of NSAIDs, yes % (n) 22.6 (7) 35.5 (11) 41.9 (13) 0.39

Total number of medication, mean (SD) 5.57 (3.73) 6.30 (3.87) 7.00 (3.89) 0.002

Physical activity % (n) 0.39

low 19.7 (47) 22.4 (54) 23.9 (57)

medium 39.7 (95) 44.4 (107) 42.9 (102)

high 40.6 (97) 33.2 (80) 33.2 (79)

Smoking status % (n) 0.73

never smoker 36.0 (86) 38.0 (92) 29.5 (71)

current smoker 6.3 (15) 4.5 (11) 5.4 (13)

former smoker 57.7 (138) 57.4 (139) 65.1 (157)

Current alcohol intake, yes % (n) 59.8 (143) 61.8 (149) 63.3 (152) 0.4

Attrition over 5 years % (n) 0.31

completed the study 46.5 (112) 39.7 (96) 41.9 (101)

dropped out 53.5 (129) 60.3 (146) 58.1 (140)

_____________________________________________________________________________________________________________________________

*Component 3: hsCRP-related component*

*n* 241 241 242

Sex, men % (n) 38.4 (93) 41.1 (99) 40.7 (98) 0.82

Occupational class 0.08

routine/manual 48.1 (113) 45.6 (104) 58.1 (132)

intermediate 14.5 (34) 16.7 (38) 12.3 (28)

higher managerial/administrative 37.4 (88) 37.7 (86) 29.5 (67)

Marital status % (n) 0.75

single 7.4 (18) 9.2 (22) 9.6 (23)

widowed/separated/divorced 61.2 (148) 57.9 (139) 62.1 (149)

married 31.4 (76) 32.9 (79) 28.3 (68)

Self-rated health % (n) <0.001

excellent/very good 46.1 (111) 43.0 (102) 32.8 (77)

good 37.3 (90) 39.2 (93) 36.2 (85)

fair/poor 16.6 (40) 17.7 (42) 31.1 (73)

BMI % (n) 0.001

<18.5 9.7 (23) 5.2 (12) 5.3 (11)

>18.5<25 54.7 (129) 54.7 (127) 45.6 (94)

>25<30 30.5 (72) 32.8 (76) 31.6 (65)

>30 5.1 (12) 7.3 (17) 17.5 (36)

Fat mass, kg mean (SD) 17.65 (7.05) 18.57 (7.04) 20.20 (8.98) 0.02

Fat-free mass, kg mean (SD) 44.00 (8.88) 45.46 (9.36) 46.60 (8.72) 0.002

Height, cm 161.69 (7.18) 161.71 (7.62) 161.13 (8.10) 0.51

Waist circumference, cm mean (SD) 89.00 (11.52) 90.77 (11.91) 93.10 (12.61) 0.001

GDS % (n) 0.001

no depression 83.7 (195) 81.0 (187) 70.1 (155)

mild depression 8.6 (20) 13.9 (32) 16.3 (36)

severe depression 7.7 (18) 5.2 (12) 13.6 (30)

Dementia, yes % (n) 4.1 (10) 7.1 (17) 11.6 (28) 0.007

Arthritis in hands, yes % (n) 33.3 (16) 20.8 (10) 45.8 (22) 0.07

Cardiovascular diseases % (n) 0.006

none (0) 25.6 (62) 23.7 (57) 26.6 (64)

1-2 69.0 (167) 68.5 (165) 58.9 (142)

3-4 5.4 (13) 7.9 (19) 14.5 (35)

Peripheral vascular diseases, yes % (n) 6.6 (16) 5.4 (13) 10.0 (24) 0.14

Metabolic syndrome, yes % (n) 24.0 (58) 24.1 (58) 34.0 (82) 0.02

Multi-morbidity, mean (SD) 2.15 (1.21) 2.33 (1.25) 2.33 (1.25) 0.18

Lipid profile

Total cholesterol, mmol/L mean (SD) 4.92 (1.27) 5.01 (1.25) 4.68 (1.17) 0.008

LDL cholesterol, mmol/L mean (SD) 2.71 (1.06) 2.83 (1.09) 2.63 (0.99) 0.11

HDL cholesterol, mmol/L mean (SD) 1.60 (0.43) 1.53 (0.38) 1.37 (0.38) <0.001

Total cholesterol/HDL ratio 3.17 (0.77) 3.41 (1.04) 3.56 (1.04) <0.001

Intake of NSAIDs, yes % (n) 41.9 (13) 35.5 (11) 22.6 (7) 0.39

Total number of medication, mean (SD) 6.06 (3.51) 6.27 (3.88) 6.73 (4.15) 0.16

Physical activity % (n) <0.001

low 14.2 (34) 17.2 (41) 34.6 (83)

medium 41.0 (98) 44.8 (107) 41.3 (99)

high 44.8 (107) 38.1 (91) 24.2 (58)

Smoking status % (n) 0.34

never smoker 36.8 (89) 36.4 (87) 30.3 (92)

current smoker 3.7 (9) 6.3 (15) 6.2 (15)

former smoker 59.5 (144) 57.3 (137) 63.5 (153)

Current alcohol intake, yes % (n) 62.1 (149) 62.9 (151) 60.0 (144) 0.8

Attrition over 5 years % (n) <0.001

completed the study 52.5 (127) 46.5 (112) 29.0 (70)

dropped out 47.5 (115) 53.5 (129) 71.0 (171)

______________________________________________________________________________________________________________________________

GDS, Geriatric Depression Scale; hsCRP, high sensitivity C-related protein; NSAIDs, non-steroidal anit-inflammatory drugs.

^a^One-way ANOVA (with Tukey HSD *post hoc* or Games-Howell if Levene statistics α<0.05) for normally, Kruskal-Wallis test for non-normally distributed continuous variables, and Chi-square test for categorical variables.
